# Individualized 3D-printed applicators for magnetic resonance imaging-guided brachytherapy in nasal vestibule cancer

**DOI:** 10.1016/j.phro.2024.100629

**Published:** 2024-08-17

**Authors:** Mischa de Ridder, Milena Smolic, Maarten Kastelijns, Samantha Kloosterman, Stefan van der Vegt, Johannes A. Rijken, Ina M. Jürgenliemk-Schulz, Homan Dehnad, Petra S. Kroon, Marinus A. Moerland

**Affiliations:** a Department of Radiation Oncology, UMC Utrecht, Utrecht, the Netherlands; b Department of Head and Neck Surgical Oncology, UMC Utrecht, Utrecht, the Netherlands

**Keywords:** Brachytherapy, head and neck cancer 3D-print, Nasal vestibule cancer

## Abstract

**Background and purpose:**

Brachytherapy is treatment of choice for early stage nasal vestibule cancer. Over the years improvements were achieved by means of image guided target definition, interstitial implant techniques and also individual mold techniques. The aim of this study was to improve the technique of the implant so that the need for interstitial catheters can be limited by making use of patient individualized 3D-printed applicators.

**Materials and Methods:**

In 19 patients 3D-printed applicators were used to deliver pulse dose rate (PDR) brachytherapy. All patients underwent computed tomography (CT) and magnetic resonance imaging (MRI). A pre-plan with tumor delineation and manually optimized catheter positions to achieve tumor coverage was made. Based on the pre-plan a 3D-printed applicator was manufactured. Dose was evaluated by several indices: Conformity Index, Healthy Tissues Conformity Index, Dose Homogeneity Index, Dose non-uniformity ratio, Conformal index and high dose (HD) index.

**Results:**

A high target coverage was achieved, with a median V100%_CTV_ of 99.1 % (range, 81.8–100 %) and median CI of 0.99 (range, 0.82–1.00), as well as a median V0.7Gy_GTV_ of 100 % (range, 93.0–100 %). The median HD was 0.39 (range, 0.20–0.83). Interstitial catheters were needed in 12 patients. None of the patients developed grade ≥ II toxicity within the median follow up of 18 months.

**Conclusions:**

This study shows that using 3D-printed applicators limits the need for interstitial catheters and also limits the high doses in normal tissue.

## Highlights

• 3D printed applicators are safe and feasible for nasal vestibule cancer brachytherapy.
• Use of 3D printed individual applicators cause less need for interstitial catheters.
• Toxicity with 3D printed applicators nasal vestibule brachytherapy is very limited.

## 1 Introduction

Brachytherapy is an organ-preserving treatment option in the management of squamous cell carcinomas in the head and neck region and can be used as primary treatment, sequential boost to external beam radiotherapy or as adjuvant therapy after surgery [1]. Primary brachytherapy is indicated in early stage cancers and easily accessible subsites like the lip, pinna or nasal vestibule, with the nasal vestibule being the most commonly implanted subsite [2], [3], [4]. The treatment outcomes of primary brachytherapy for cancer of the nasal vestibule (CNV) are excellent in oncological, functional and aesthetic terms [2], [5], [6], [7], [8]. Therefore brachytherapy is considered standard of care for CNV [9].

Over the years, considerable advancements have been made in brachytherapy techniques. Image-guidance is among one of the biggest improvements, allowing for better target definition and organ-at-risk (OAR) sparing. Most of the techniques are developed within the context of gynecological and prostate cancer and then translated to head and neck brachytherapy. Computed tomography (CT) is most commonly used in brachytherapy, but in recent years the use of magnetic resonance imaging (MRI) has steadily increased [10].

Implantation techniques have evolved as well as imaging techniques. Alongside traditional implantation methods (like the Paris system) more sophisticated implantation techniques like anatomical implantation are used [11]. Also, 3D dose calculation using modern treatment planning systems with dwell time optimization changed the field. Mold techniques for both surface and interstitial brachytherapy have also improved. Nonetheless, interstitial catheters cause tissue damage and also (very) high doses are delivered in normal tissue around the interstitial catheters. The use of a 3D-printer allows more degrees of freedom in catheter placement possibly enhancing usability in more patients. For superficial brachytherapy of skin cancer the use of individualized 3D printed applicators showed promising results[12], [13], [14]. Literature on 3D-printed applicators is scarce for head and neck brachytherapy limited to case reports [15] and a series reporting on 3D-printed templates for guiding I-125 seed implantation in head and neck cases [16].

A 3D-printed applicator technique for nasal vestibule brachytherapy was developed using 3D-printed biocompatible material. The aim of this study was to develop an implant technique for nasal vestibule cancer brachytherapy that minimizes the need for interstitial catheters while preserving adequate dose coverage and dose distribution.

## 2 Materials and methods

### 2.1 Patient and treatment characteristics

A chart review was performed of all patients treated at our institute with brachytherapy for a tumor in the head and neck region with the use of an individualized 3D-printed applicator. The development of this 3D-printing procedure started in 2018. From 2021 onward the same method and material (Biomed Clear resin; Formlabs, Somerville, USA) was used, thus patients in this study were included from 2021. From 2021 until 2023 in total 19 patients with squamous cell carcinoma of the nasal vestibule were treated with 3D-printed individualized applicator based brachytherapy. Of these, 17 patients had a cT1 tumor, 1 patient a cT2 tumor and 1 patient had a cT3 tumor (according to Wang et al [17]). Median follow-up of the cohort was 18 months.

The material used for the applicator (Biomed Clear) has a relative electron density of 1.1 and a CT number of 100 Hounsfield units (HU)[18]. Therefore, it is water equivalent and does not perturb the dose calculations. Furthermore, the material was found to be stable over a period of at least 30 days when exposed to water, radiation and heat.

The treatment outcome and toxicity were scored. Outcome was clinically determined at the most recent moment of follow-up. This study was approved by our institutional review board (20–519/C).

### 2.2 Treatment workflow

**Fig. 1:**
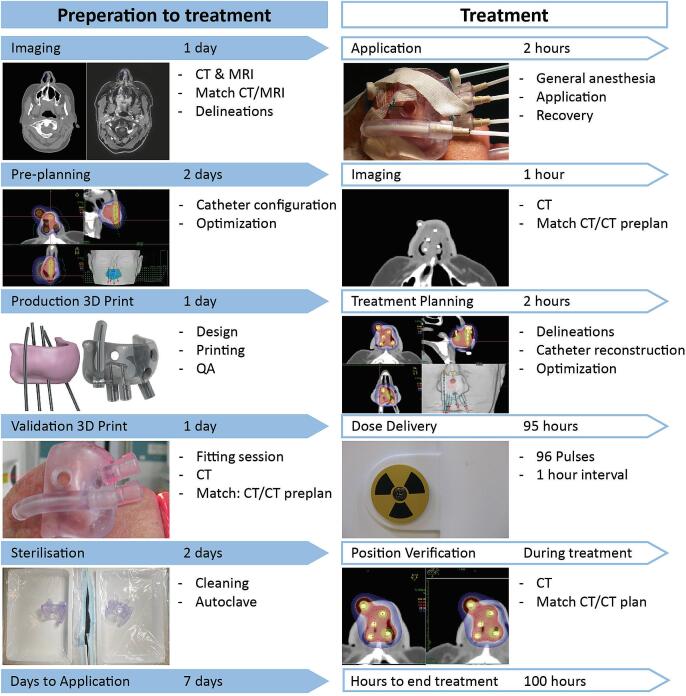
An overview of the workflow, including the time needed for each step.

The workflow of the treatment included pre-treatment imaging, delineation, calculation of a pre-plan and design of the 3D-applicator, 3D-printing of the applicator, a fitting session with the patient, placement of the applicator and insertion of interstitial catheters, CT-based simulation, treatment planning and dose delivery. Fig. 1 shows an overview of the workflow from preparation to treatment. All imaging, materials and equipment used in these workflows is presented in Table 1.

**Table 1:** Summary of equipment.

**Equipment**	**Details**
Catheters	Flexible Button End (6Fr flexible implant tubes; Elekta, Stockholm, Sweden) − Flexible Blind End (6Fr flexible implant tubes; Elekta, Stockholm, Sweden) − ProGuide (6Fr ProGuide catheters; Elekta, Stockholm, Sweden)
Lock inserts	Lock Insert (Elekta, Stockholm, Sweden)
3D printer	Formlabs 3B+and Formlabs 3B (Formlabs, Somerville, USA)
3D printed material	BioMed Clear (Formlabs, Somerville, USA)
Afterloader	MicroSelectron-V3 PDR (Elekta, Stockholm, Sweden)
CT-scanner	Philips Brilliance Big Bore v3.6 and v4.8 (32 slice)
MRI-scanner	Philips Ingenia 3 T

For pre-treatment imaging CT-imaging was performed on a Philips Big Bore scanner (v3.6 and v4.8) with 1 mm slices. MRI was performed on a 3 T Ingenia Philips scanner in prone position using a dedicated head and neck coil. The MRI scan protocol included a T2 weighted sequence (T2 TSE mDIXON), T1 weighted sequence with (T1 3D TFE gd) and without gadolinium (T1 TSE), and diffusion weighted sequences (DWI SPLICE and ADC SPLICE).

For every patient a pre-plan was made starting with target delineation for each individual patient. The gross tumor volume (GTV) was delineated based on findings from physical examination, CT scan and MRI. The clinical target volume (CTV) was contoured according to ESTRO and GEC-ESTRO guidelines [19], [20], by adding a uniform margin of 5 mm around the GTV cropped to anatomical boundaries and air cavities. Treatment planning was done using Oncentra Brachy (version 4.5 – 4.6) (Elekta, Stockholm, Sweden). A pre-plan was simulated with manually reconstructed catheter positions. Optimization of treatment dwell times was performed by constraining the coverage of the GTV by the 0.7 Gy isodose and CTV with at least 0.6 Gy (100 %) isodose. This process was done in three steps, started with a normalization with a 5 mm box, second step was a graphical optimization and last step was manual optimization. Confluence of 200 % isodose regions was avoided. Depending on the subsite, maximum point dose to OAR was taken into consideration during optimization. Dwell position spacing used in the treatment plans was 5 mm. The interval between pulses was fixed at 60 min and pulses were scheduled for every hour of the day and night. Patients were scheduled to start on Mondays so that treatment was finished on Fridays.

Next step in the workflow was the design of the applicator. A basic applicator volume was modelled in Oncentra Brachy by extracting an expanded skin contour of + 5 mm minus an expanded skin contour of + 1 mm. The structure file (RTstruct) was converted to STL files using open source software Slicer (version 4.10.2). Catheter coordinates were converted to a CSV file per catheter using Excel (Microsoft 2016). For the applicator design the STL and CSV files were combined using Fusion360 (Autodesk). Catheter paths and counter of negative lock inserts were added to the basic applicator volume to fix the catheters to the 3D-printed applicator. To fixate the applicator to the skin, stitching holes were added. For patient comfort, air holes were made if nostrils were wide enough.

The applicator model was prepared for 3D-printing with a Formlabs printer using print preparation software Preform (Formlabs, Somerville, USA). Catheter paths and counter of negative lock inserts in the applicator were positioned as vertically as possible. The printing was done with a density of 1 g/cm^3^ (solid). After printing, the applicator was washed in 99.5 % isopropyl alcohol (IPA) for 20 min using a Form Wash (Formlabs, Somerville, USA) and 5 min in a clean bath of IPA (99.5 %). The applicator was cured for 60 min at 60 ^0^C using the Form Cure (Formlabs, Somerville, USA). Supports were removed and finished by hand.

After printing of the model a test session was scheduled for all patients to insure an optimal fit before performing definitive applicator placement and, if indicated, additional interstitial needles under general anesthesia. Part of this test session was a CT-simulation with the 3D-printed applicator to evaluate fit and possible air gaps between the applicator and the skin of the patient.

The applicator placement was done under general anesthesia with orotracheal intubation. Button ends were used to fixate interstitial catheters, while intracavitary ProGuide catheters (Elekta, Stockholm, Sweden) were fixated inside the 3D-printed applicator with lock inserts. On seldom occasion flexible blind end catheters (Elekta) were used. The 3D-printed applicator was fixated to the patient with non-absorbable sutures. If indicated, patients received prophylactic antibiotics.

After applicator placement the patient underwent a second CT-scan. This CT-scan was used to visualize the definitive application of the applicator and interstitial catheters (if used). This second CT-scan was registered to the pre-plan CT-scan, and pre-plan delineations were adjusted on this CT-scan. Catheter reconstruction was performed manually in Oncentra Brachy.

The prescribed pulse dose was 0.6 Gy each hour (day and night), with number of pulses ranging from 74 to 97. The pre-plan was used as a starting point for the definitive treatment plan. Based on imaging and catheter reconstruction the pre-plan was adjusted to fulfill clinical goals as described above. During the five days of treatment, patients were admitted at the radiation ward and connected to the pulsed-dose-rate (PDR) afterloader (microSelectron, Elekta, Sweden).

All patients were treated within one working week and approximately halfway through the treatment, all patients underwent a position verification CT-scan in order to be able to adapt the treatment plan in case of excessive swelling or catheter displacements.

### 2.3 Dose evaluation

The indices for dose evaluation were derived from definitions according to literature [21], [22], [23]. Due to the relatively large portion of the treated volume being in applicator or air, the V100%_Implant_ was calculated for tissue only by excluding applicator and air from the volume covered by the 100 % isodose. In order to quantify how much of the high dose regions in the treated volume was captured by applicator and air, an additional high dose parameter (HD) was calculated as HD = (V200%_applicator_ + V200%_air_)/V200%_implant_. EQD2 values were calculated for the total number of pulses with a T_1/2_ value of 1,5 h and an α/β value of 10 Gy for target and 3 Gy for OARs.

### 2.4 Follow-up

Toxicity was scored according to the common terminology criteria for adverse events (CTCAE) version 4.0.Patients underwent response evaluation with clinical examination at week 6 after treatment accompanied by MRI at 3 months after treatment. The standard follow-up schedule for patients in case of complete response is every 2 months in the first year, every 3 months in the second year, every 4 months in the third year and every 6 months up to 5 years after treatment. Since patients were included from 2021 the early patients were in year 3 of their follow-up. These follow-up appointments consisted of clinical examination of nose and neck nodes. In case of clinical suspicion additional cross sectional imaging was performed.

Survival, local and regional control was estimated using the Kaplan Meier method.

## 3 Results

A high target coverage was achieved, with a median V100%_CTV_ of 99.1 % (range, 81.8–100 %) and median CI of 0.99 (range, 0.82–100), as well as a median V0.7Gy_GTV_ of 100 % (range, 93.0–100 %). This coverage was achieved using between 3 and 8 catheters. In seven patients there was no need for additional interstitial catheters, 11 patients needed one interstitial catheter and in one patient it was necessary to place two interstitial catheters. The proportion of healthy tissue receiving a dose greater than or equal to 100 % of the prescribed reference dose, described by the index HTCI, had a median value of 0.37 (range, 0.15–0.64) (Table 2). A combination of these two indices is reflected in the COIN, which had a median value of 0.35 (range, 0.14–0.58).

**Table 2:** An overview of the dose/volume planning metrics.

	**Median**	**Range**
**GTV**		
Volume (cm^3^)	0.3	0.1–3.4
D98% (Gy) per pulse	0.9	0.6 – 1.1
D98% EQD2_α/β=10 Gy_ (Gy)	91.5	63.5–134.6
V0.7 Gy (%)	100.0	93.0–100.0
**CTV**		
Volume (cm^3^)	2.1	0.5–7.3
D90% (Gy) per pulse	0.7	0.5 – 0.8
D90% EQD2_α/β=10 Gy_ (Gy)	70.4	48.0–83.5
D98% (Gy) per pulse	0.6	0.4 – 0.7
D98% EQD2_α/β=10 Gy_ (Gy)	61.3	34.9–71.7
V0.6 Gy (%)	99.1	81.8–100.0
**OAR**		
Upper lip		
D2cm^3^ (Gy) per pulse	0.1	0.0 – 0.2
D2cm^3^ EQD2_α/β=3 Gy_ (Gy)	4.8	2.7–17.8
Bone		
D2cm^3^ (Gy) per pulse	0.2	0.1 – 0.4
D2cm^3^ EQD2_α/β=3 Gy_ (Gy)	14.5	5.1–28.9
**Implant and source**		
V100% _Implant_ (cm^3^)	11.2	4.7–38.5
V150% _Implant_ (cm^3^)	4.9	2.2–14.7
V200% _Implant_ (cm^3^)	2.3	1.1–6.7
TRAK (μGy@1m)	84.0	46.0–186.0
Treatment time/pulse (sec)	60.6	27.2–201.9
**Indices**		
CI	0.99	0.82–1.00
HTCI	0.37	0.15–0.64
DHI	0.41	0.18–0.66
DNR	0.59	0.34–0.82
COIN	0.35	0.14–0.58
HD	0.39	0.20–0.83

*Dx = dose received by x% of the target volume; Vy = volume receiving y% of the prescribed dose;*

*CI=Conformity Index = V100%_CTV_/V_CTV_; HTCI=Healthy Tissues Conformity Index = V100%_CTV_/(V100%_Implant_ −V100%_applicator_ −V100%_air_); DHI=Dose Homogeneity Index = (V100%_CTV_ – V150%_CTV_)/ V100%_CTV_; DNR=Dose non-uniformity ratio = V150%_CTV_/ V100%_CTV_; COIN=Conformal index = CI x HTCI=V100%_CTV_^2^/ (V_CTV_ x (V100%_Implant_ −V100%_applicator_ −V100%_air_)); HD = (V200%_applicator_ + V200%_air_)/V200%_Implant_*.

The median D98%_CTV_ achieved was 61.3 Gy EQD2_α/β=10 Gy_ (range, 34.9–71.7 Gy EQD2_α/β=10 Gy_), while the median D98%_GTV_ was 91.5 Gy EQD2_α/β=10 Gy_ (range, 63.5–134.6 Gy EQD2_α/β=10 Gy_). The median D2cm^3^_upper lip_ was 4.8 Gy EQD2_α/β=3 Gy_ (range, 2.7–17.8 Gy EQD2_α/β=3 Gy_), while the median D2cm^3^_bone_ was 14.5 Gy EQD2_α/β=3 Gy_ (range, 5.1–28.9 Gy EQD2_α/β=3 Gy_). The median HD was 0.39 (range, 0.20–0.83).

Local complete remission was achieved in 18/19 of the patients. Estimated local control with Kaplan Meier method was 94 %. The one patient with local recurrent disease developed a recurrence one year after treatment. The patient was staged with a cT3 tumor. Also, one patient developed a regional recurrence occurred within the first year after treatment. This patient underwent salvage bilateral neck dissection (pN2c) with adjuvant external beam radiotherapy to the neck. All patients were free of disease until sensor date (March 2024).

The overall survival was estimated 95 %. Overall, no grade II or higher toxicity was scored within the patient cohort during follow-up.

## 4 Discussion

This study showed that with a pre-plan, 3D-printed individual applicator, image guided brachytherapy technique it is possible to decrease the need for interstitial catheters substantially. In this study the need for interstitial needles is limited to a median of one interstitial needle and a maximum of only two needles. Compared to other series this is a major reduction: Czerwinski [2] et al. reported a range from 4 to 21 interstitial catheters, Levendag et al [24] reported 3–7 interstitial catheters and Tagliaferri et al [6] reported 5–18 catheters. In our opinion, less interstitial catheters results in less tissue damage. The possible drawback of using less interstitial catheters is a higher dose to the surface. However, doses around interstitial catheters are by definition (very) high, and with customized and pre-plan optimized applicators is was possible to capture the high dose as much as possible in the applicator. With this the dose to the uninvolved mucosa and skin can be limited. The oncological outcome achieved with this approach are comparable to our historical [7] and other brachytherapy series [6], [8], [24], although the follow-up period is short to draw firm conclusions.

Choice of material is crucial for a successful applicator design. The design and production process of the applicators were monitored and evaluated with consideration for the fact that the applicators are a Medical Device of risk class IIb according to the Medical Device Directive [25].The material BioMed Clear (Formlabs, Sommerville, USA) fulfilled the criteria for applicator design. This hard and strong material is biocompatible for long-term skin and mucosa contact, FDA approved for healthcare applications and compatible with modern sterilization methods. The printed BioMed Clear material retains its shape under the influence of body temperature, fluid and irradiation.

3D-printed patient individual applicators have previously been described for skin [12], [13], [14], cervical [26], [27] and vaginal [28], [29], [30] brachytherapy. For skin lesions it was found that 3D-printed applicators improved applicator geometry and dose optimization. For instance, while maintaining coverage of the CTV, the dose to the patient’s surface was less than 150 % [13]. For vaginal brachytherapy it was found that with 3D-printed applicators it was possible to deliver higher doses to larger volumes compared to multichannel cylinders without compromising OAR dose [26]. Data on toxicity and late morbidity is still lacking. Another group evaluated 3D-printed individual applicators for advanced gynecological cancers and found that it was possible to improve dose to the high risk CTV without compromising OAR dose [27].

The use of 3D-printing in brachytherapy is a promising development in the field which has many potential applications in future. The large number of degrees of freedom and flexibility inherent to the technique allows individual and optimal dose optimization. However, delineation guidelines and dose optimization guidelines are essential to boost the progress. The GEC-ESTRO guideline [4], [19] provides some assistance regarding this, but for accurate comparisons and also possible improvements of applicators there is a need for standardized reporting on dose and applicator parameters.

Besides optimized treatment, the use of personalized applicators makes it possible to improve patient experience during treatment. Individualized applicators are specifically designed to fit patient anatomy optimally. For example, we applied small nostril holes in the applicator for patients to be able to breathe through their nose or insert nasal spray, and we designed a small gutter in a nasopharynx applicator for the nasogastric tube.

The 3D-printed applicators also allow for extra material to be printed on parts of the applicator to shield the healthy mucosa, such as an uninvolved septum, from the 200 % or 150 % isodose.In conclusion, 3D-printed applicators for CNV brachytherapy resulted in adequate dose distributions with minimum need for interstitial catheters. Short term clinical outcome in terms of local control and toxicity is excellent. Uniform reporting is necessary in order to compare different methods in future.

## Declaration of Competing Interest

The authors declare that they have no known competing financial interests or personal relationships that could have appeared to influence the work reported in this paper.
